# TGFβ/Activin signalling is required for ribosome biogenesis and cell growth in *Drosophila* salivary glands

**DOI:** 10.1098/rsob.160258

**Published:** 2017-01-25

**Authors:** Torcato Martins, Nadia Eusebio, Andreia Correia, Joana Marinho, Fernando Casares, Paulo S. Pereira

**Affiliations:** 1 I3S-Instituto de Investigação e Inovação em Saúde, Universidade do Porto, Porto 4150-180, Portugal; 2 Instituto de Biologia Molecular e Celular (IBMC), Universidade do Porto, Porto 4150-180, Portugal; 3 Cell Cycle Development Group, Department of Genetics, University of Cambridge, Downing Street, Cambridge CB2 3EH, UK; 4 Centro Andaluz de Biología del Desarrollo (CABD), CSIC-JA-Universidad Pablo de Olavide. Ctra. de Utrera km1, Seville 41013, Spain

**Keywords:** *Drosophila*, cell growth, Punt (Put), TGFβ/Activin, nucleolus, ribosome

## Abstract

Signalling by TGFβ superfamily factors plays an important role in tissue growth and cell proliferation. In *Drosophila*, the activity of the TGFβ/Activin signalling branch has been linked to the regulation of cell growth and proliferation, but the cellular and molecular basis for these functions are not fully understood. In this study, we show that both the RII receptor Punt (Put) and the R-Smad Smad2 are strongly required for cell and tissue growth. Knocking down the expression of Put or Smad2 in salivary glands causes alterations in nucleolar structure and functions. Cells with decreased TGFβ/Activin signalling accumulate intermediate pre-rRNA transcripts containing internal transcribed spacer 1 regions accompanied by the nucleolar retention of ribosomal proteins. Thus, our results show that TGFβ/Activin signalling is required for ribosomal biogenesis, a key aspect of cellular growth control. Importantly, overexpression of Put enhanced cell growth induced by *Drosophila* Myc, a well-characterized inducer of nucleolar hypertrophy and ribosome biogenesis.

## 1. Introduction

Tissue growth is a very complex process that requires interplay between multiple signalling pathways to ensure that an organ achieves its proper size and shape. Transforming growth factor beta (TGFβ) and bone morphogenetic protein (BMP) signalling pathways play multiple roles during animal development through the regulation of cellular growth, proliferation, differentiation and survival [1]. At the cell surface, the secreted polypeptides of the TGFβ superfamily (TGFβ, BMP, Activin and Nodal) bind tetrameric complexes of type I (RI) and type II (RII) serine/threonine kinase receptors. Ligand binding allows the active RII receptors to phosphorylate serines and threonines within the GS domain of RI receptors, which in turn phosphorylate and activate Smads. Receptor-activated (R) Smads then associate with the common-mediator (Co) Smad and the complex is shuttled to the nucleus where it regulates target gene expression [1]. TGFβ signalling can either suppress or promote cell growth and proliferation, a phenomenon described as the TGFβ paradox in the context of cancer progression [2,3]. TGFβ is also an important promoter of epithelial–mesenchymal transition (EMT), where its activity leads to increased protein synthesis and cell size through activation of the PI3K, Akt and mTOR complex 1 [4]. Furthermore, the activity of TGFβ receptor I kinase was shown to be required for glucose-induced hypertrophy in both fibroblasts and epithelial cells [5]. Similar to high glucose, adding TGFβ to these cells caused an increase in protein synthesis and cell size [5]. In a subsequent study, treatment with the anti-TGFβ1 neutralization antibody (1D11) was shown to protect mice from obesity and diabetes [6]. Thus, the control of cell growth by TGFβ in different cell types and contexts is expected to play important roles in diabetes and cancer pathology.

The TGFβ pathway is evolutionarily conserved in *Drosophila*, where both the BMP and TGFβ/Activin branches are crucial regulators of developmental processes [7]. Put is a common RII receptor for both signalling branches, and it heterodimerizes with branch-specific RI receptors to ensure pathway specificity. In the TGFβ/Activin branch, Put binds the RI receptor Baboon (Babo) that phosphorylates Smad2 (also known as Smox) in response to the Activin-β (Actβ), Dawdle (Daw) and Myoglianin (Myo) ligands [8–11]. The TGFβ/Activin pathway was shown to regulate axonal outgrowth and remodelling [12–14], as well as proliferation of neuroblasts and wing imaginal disc cells [8,9,15].

In a recent eye-targeted double-RNAi screen, we identified a genetic interaction between several *Drosophila* TGFβ signalling members (including Put, Baboon and Smad2) and the nucleolar regulator Viriato (Vito)/Nol12 [16]. Previously, we had shown that Vito acts downstream of dMyc to ensure a coordinated nucleolar response during dMyc-stimulated growth [17]. Thus, Vito could play a role in dMyc-mediated increase in the rate of ribosome biogenesis in the nucleolus, one of the main mechanisms by which dMyc drives growth [18]. Because the mechanisms enabling TGFβ signalling to induce cell growth and proliferation are poorly understood, we pursued the analysis of the novel link between TGFβ signalling and nucleolar-based events. Here, we study the cell-autonomous functions of TGFβ/Activin signalling in cell growth, using the salivary gland as a model tissue. During larval stages, the salivary gland is an endoreplicative tissue where overall growth correlates directly with cell growth, and that allows easy characterization of subcellular structures.

## 2. Results

### 2.1. TGFβ/Activin signalling is required for tissue growth and nucleolar dynamics

**Figure 1.**
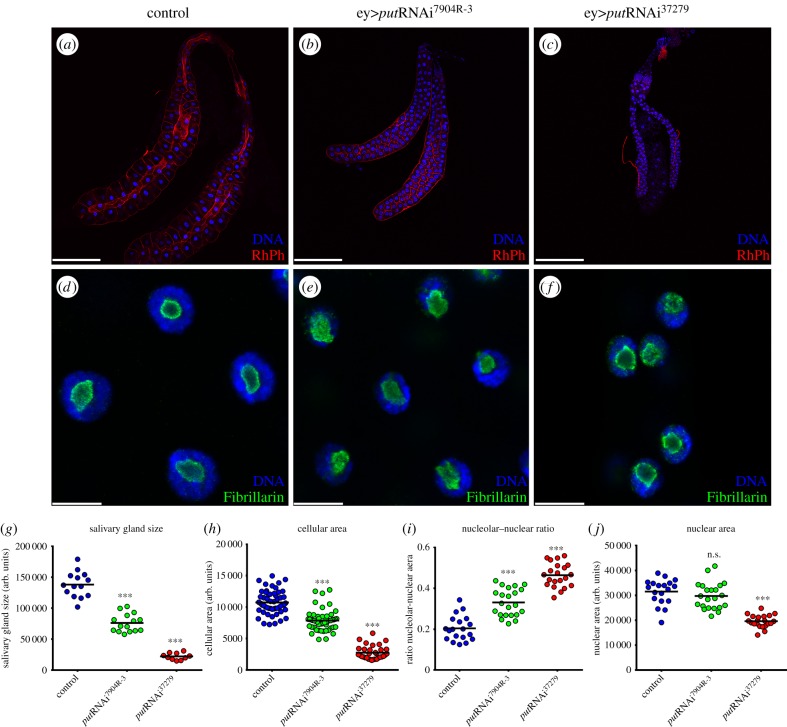
TGFβ/Activin signalling is required for tissue growth and nucleolar dynamics. (*a–c*) Salivary glands show a substantial reduction in overall size upon *put* depletion. Low magnifications images of salivary glands from third-instar *Drosophila* larvae expressing (*a*) UAS-*lacZ* (control), (*b*) UAS-*put*RNAi^7904R-3^ and (*c*) UAS-*put*RNAi^37279^ under the control of the *ey-Gal4* driver. Salivary glands of the indicated genotypes were stained for the cell limits with RhPh (red) and counterstained with DAPI (blue). (*d–f*) Put requirement for salivary glands growth is linked to an increase in nucleolar size. (*d*) Localization of the nucleolar marker Fibrillarin in control nuclei of salivary glands. (*e*) *put* RNAi^7904R-3^ results in ectopic accumulation of Fibrillarin. (*f*) Strong *put*RNAi^37279^ induction results in expansion of the Fibrillarin at the nucleolus. (*g*) Scatter plot representative of *put* requirement for salivary gland growth. (*h*) Salivary gland growth deficit is linked to a decrease in the cellular area. (*i*) *put* depletion causes an increase in nucleolar/nuclear area ratio in the salivary glands. (*j*) Scatter plot showing nuclear area quantification of the described genotypes (*n* = 25–40; ***p < 1 × 10^–4^). Scale bars: (*a–c*) 200 µm, (*d–f*) 20 µm.

To study possible cell-autonomous functions of TGFβ signalling in salivary gland cell growth, we downregulated the expression of the RII receptor Put (figure 1). RNA interference (RNAi) was targeted to the post-mitotic salivary glands and eye imaginal discs using the ey-Gal4 driver [17]. The two *put*RNAi lines we used (VDRC^37279^ and NIG-FLY^7904R-3^) target non-overlapping regions of *put* and inhibited the progression of photoreceptor differentiation in the eye imaginal disc (electronic supplementary material, figure S1*a–c*). This phenotype mimics mutant *put* phenotypes [19], and confirms the specificity and efficiency of the RNAi knockdown. Importantly, knocking down *put* expression in the salivary glands caused a strong reduction in cellular area (figure 1*a–c*,*h*) with a strong effect on tissue growth (figure 1*g*). In particular, expression of the stronger *put*RNAi^37279^ caused a significant decrease in salivary gland area (84%, *p* < 1 × 10^−4^; figure 1*c,g*), and completely inhibited the onset of the photoreceptor differentiation (electronic supplementary material, figure S1*c*). Our recent work established a genetic interaction between *put* and *vito*, which encodes for a regulator of nucleolar organization and tissue growth [16]. Because uncoordinated nucleolar hypertrophy has been associated with defective cell growth [17,20], we evaluated whether Put controlled nucleolar dynamics. We stained salivary glands with anti-Fibrillarin, a nucleolar protein involved in pre-rRNA processing, or anti-AH6 to label nucleoli and DAPI for DNA (figure 1*d–f*, and not shown). Knocking down *put* caused an expansion of the nucleolar area, reflected in an increased ratio between nucleolar and nuclear areas. Expression of *put*RNAi^7904R-3^ resulted in a 62% increase in the nucleolar/nuclear area ratio (*p* < 1 × 10^−4^), whereas expression of *put*RNAi^37279^ caused an even stronger increase (127%, *p* < 1 × 10^−4^; figure 1*i*). Thus, for *put* depletion in salivary glands, we observe a direct correlation between increased nucleolar/nuclear ratio and diminished tissue growth (*R*^2^ = 0.99468). This suggests that *put* controls nucleolar dynamics during salivary gland cell growth. Importantly, the weaker *put*RNAi^7904R−3^ did not significantly affect nuclear size (figure 1*j*), suggesting that the reduced nuclear size caused by the stronger *put*RNAi^37279^ (figure 1*j*) is secondary or a consequence of the observed nucleolar alterations.

**Figure 2.**
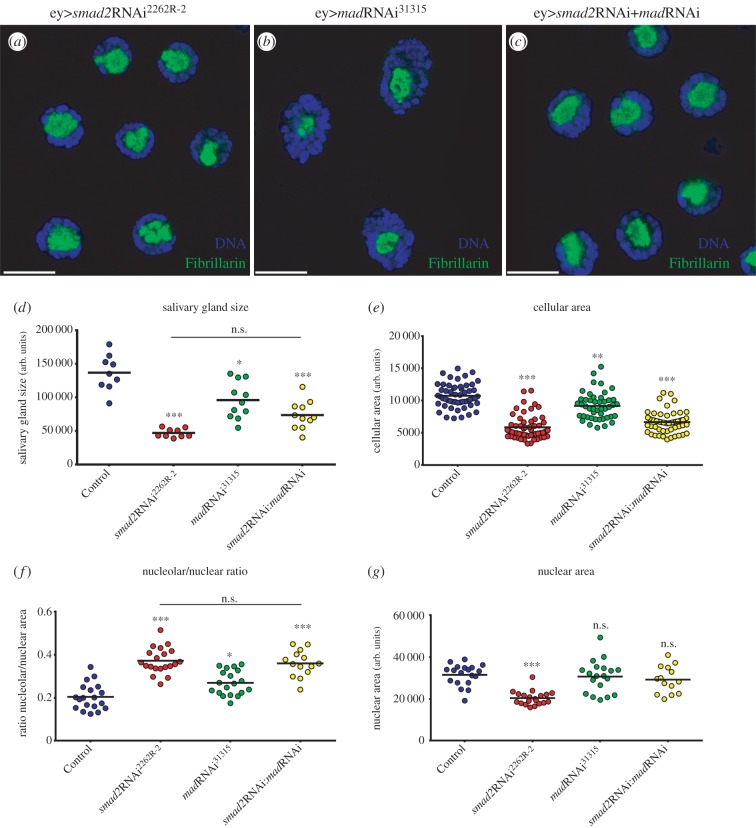
Activin R-Smad *smad2* is required for nucleolar dynamics. (*a–c*) TGFβ R-Smads contribution for nucleolar activity. Fibrillarin expansion caused by (*a*) Activin R-Smad *smad2* depletion, (*b*) BMP R-Smad *mad* depletion and (*c*) co-depletion of both R-Smads presents a similar phenotype to *smad2*RNAi. In all panels, DNA was labelled with DAPI and represented in blue. Scale bars 20 µm. (*d–g*) Scatter plots showing the quantification of the different organ and cellular parameters after R-Smads depletion. *smad2*RNAi has a stronger effect on growth levels than *mad*RNAi, both in overall tissue size (*d*) and cellular area (*e*). Reduction of the Activin pathway activity increases the relative nucleolar area (*f*) with a mild impact on the nuclear size (*g*). (*n* = 25–40; n.s. means no statistical difference between samples; *p < 0.05; **p < 0.01 and ***p < 1 × 10^–4^).

**Figure 3.**
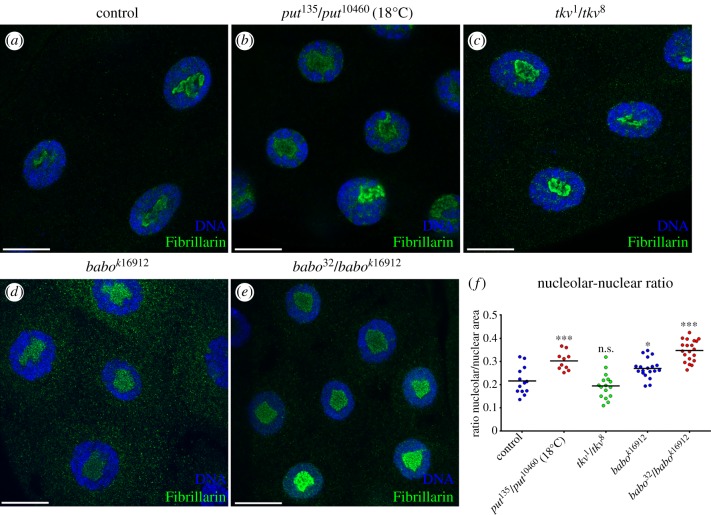
TGFβ/Activin mutants display nucleolar phenotypes. (*a–e*) Reduced Activin activity induces nucleolar expansion. (*a*) Control nuclear and nucleolar staining (*w1118*). (*b*) *put* mutant combination (*put^135^/put^10460^*) grew at a permissive temperature (18°C) and present an expansion of the nucleolar area. (*c*) Dpp receptor *tkv* mutations (*tkv^1^/tkv^8^*) do not change the nucleolar/nuclear ratio. (*d*) A weak allele for the Activin receptor type I *baboon* (*babo^k16912^*) shows a small expansion of the nucleolar area. (*e*) The heteroallelic combination of *baboon* (*babok^16912^/babo^32^*) shows a nucleolar expansion equivalent to *put* mutant combinations. All the nuclei were stained with DAPI (blue) and the nucleoli with Fibrillarin (green). (*f*) Scatter plot represents the nucleolar accumulation of the Fibrillarin in salivary glands of the indicated genotypes (*p < 0.05; ***p < 0.001). Scale bars: 20 μm.

The RII receptor Put heterodimerizes with RI receptors Tkv or Babo to mediate BMP and TGFβ/Activin signalling, respectively. To study the contribution of each signalling branch to nucleolar regulation, we knocked down the expression of the TGFβ/Activin-branch-specific R-SMAD, Smad2 [8,21], and of the BMP-branch-specific R-SMAD Mad [22,23], either alone or in combination (figure 2). To validate the efficiency and specificity of the RNAi lines for Smad2 and Mad, we targeted their expression to the developing eye imaginal disc. In agreement with previous observations, downregulation of TGFβ/Activin signalling by *smad2*RNAi affected growth of the eye disc (electronic supplementary material, figure S1*e*) [8], whereas downregulation of BMP signalling by *mad*RNAi strongly interfered with both tissue growth and patterning (electronic supplementary material, figure S1*f*) [24]. Importantly, expression of *smad2*RNAi caused a strong increase in the nucleolar/nuclear area ratio in salivary gland cells, which is not induced by *mad*RNAi expression (figure 2*f*). Furthermore, co-expression of *mad*RNAi failed to enhance the increase in nucleolar size, or the decrease in cellular and tissue size, induced by *smad2*RNAi (figure 2 and electronic supplementary material, figure S1*g–j*). In addition, salivary glands of larvae mutant for *put* or for *babo*, the Activin branch-specific RI receptor, also displayed a nucleolar phenotype, not observed in mutants for *tkv*, the BMP branch-specific RI receptor (figure 3*a–f*). Taken together, these results show that the TGFβ/Activin pathway is required for growth and normal nucleolar dynamics.

### 2.2. TGFβ/Activin is required for the coordination of the ribosome biogenesis

**Figure 4.**
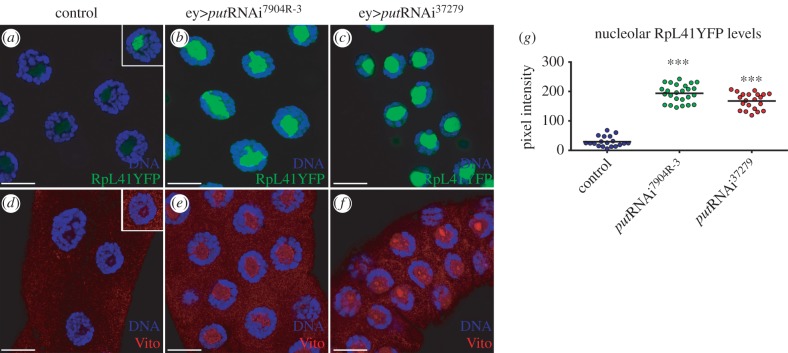
Inhibition of TGFβ/Activin signalling activity by *put*RNAi causes a strong nucleolar accumulation of RpL41 and Vito. (*a–c*) Put regulates nucleolar size and accumulation of nucleolar components. (*a*) Localization of the large ribosome subunit RpL41YFP in control nuclei salivary glands. Inset shows nuclei with higher acquisition settings to determine the precise localization of RpL41YFP. (*b*) *put* RNAi^7904R-3^ depletion results in ectopic accumulation of RpL41YFP. (*c*) Strong *put*RNAi^37279^ induction results in a growth deficit with several fold accumulation of RpL41YFP. (*d–f*) Vito accumulates at the nucleolus in *put* loss-of-function genotypes. (*d*) Vito localizes at the nucleolus in control where it strongly accumulates in (*e*) *ey* *>* *put*RNAi^7904R-3^ and (*f*) *ey* *>* *put*RNAi^37279^. In all panels, DNA was labelled with DAPI and shown in blue. (*g*) Scatter plot represents the nucleolar accumulation of the RpL41YFP protein in salivary glands of the indicated genotypes (*n* = 25–40; ***p < 1 × 10^–4^). Scale bars 20 µm.

**Figure 5.**
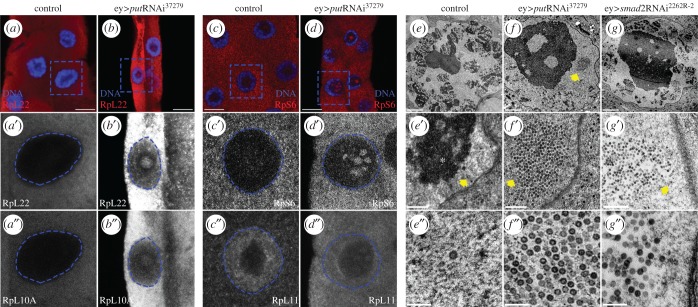
The TGFβ/Activin pathway is required for the coordination of the ribosome biogenesis. (*a–d*) Analysis of ribosome proteins (RPs) nucleolar localization. (*a–a*″) RpL22 and RpL10A are not detectable at the nucleolus in control nuclei. (*b–b*″) Depletion of *put* results in the accumulation of RpL22 and RpL10A at the nucleoplasm and nucleolus. (*c–c*″) RpS6 is not present at the nucleoplasm in control salivary glands while RpL11 has peripheral nucleoplasm localization. (*d–d*″) Decrease of TGFβ/Activin signalling results in nucleolar enrichment of RpS6 and RpL11. Blue squares represent the magnified area presented in *a′–d*″. Blue dashed circles represent the nuclear area of the respective nuclei. (*e–g*) Transmission electron micrographs of nuclear regions of independent salivary gland cells and examples of higher magnifications of the nucleoli (*e″–g*″). (*e–e*″) Higher magnifications of the control nucleoplasm reveal the nucleolus as the higher electrodense structure. Arrow points to an example of a single nuclear particle and asterisk represents the electrodense structure of the chromatin. In the absence of *put* (*f–f*″), or *Smad2* (*g–g*″), the hypertrophied nucleolus presents vacuolar-like regions and clusters with a large number of particles (arrows). Scale bars: (*a–d*) 20 µm, (*e–g*) 2 µm, (*e′–g′*) 200 nm, (*e*″–*g*″) 20 nm.

The increased size ratio of the nucleolus apparently contradicts the reduction in tissue growth observed when the TGFβ/Activin pathway is attenuated. One possibility is that the increase in size is reflecting a defective, rather than a gain of nucleolar function (e.g. a defective production of ribosomes). The nucleolus plays a major role in cell growth through the coordination of three steps in ribosome biogenesis: transcription of pre-rRNA by polymerase I, processing of pre-rRNA, and assembly of the large (60S) and small (40S) ribosome subunits [25]. Defects in the biogenesis of the large or small subunits (e.g. pre-rRNA processing deficits) lead to nucleolar stress accompanied by alterations in the localization of ribosomal proteins and other nucleolar factors [25]. To study if TGFβ/Activin signalling regulates ribosome biogenesis in the salivary gland, we analysed the localization pattern of the ribosomal protein RpL41 [26]. In the nuclei of control cells, RpL41 was mainly nucleolar restricted (figure 4*a*), as previously observed [27]. Inhibition of TGFβ/Activin signalling activity by *put*RNAi caused a strong nucleolar accumulation of RpL41 (figure 4*a–c,g*). Similarly, we also observed an increase in the nucleolar localization of Vito (figure 4*d–f*). Nucleolar enrichment of Vito was not homogeneous, as we detected intranucleolar regions with higher Vito levels (figure 4*d–f*). These results prompted us to further evaluate the role of TGFβ/Activin signalling in nucleolar structure and ribosome biogenesis. In control cells, immunostaining with αRpL22, αRpL10A and αRpS6 antibodies showed that these ribosomal proteins are mainly cytoplasmic, possibly reflecting a transient association with pre-ribosome subunits at the nucleolus (figure 5*a*–*a*″,*c*–*c*″). Interestingly, in *put*RNAi both RpL22 and Rpl10A are concentrated in the peripheral nucleoplasm and in the nucleolus (figure 5*b–b*″), whereas RpS6 accumulates in granular intranucleolar spots (figure 5*d-d*′). In comparison with control cells, RpL11 is also found at higher levels in these nucleolar granular spots, where it co-localizes with RpS6 (figure 5*d*″). This pattern of nucleolar accumulation was not a general attribute of all ribosomal proteins. RpS9 is mainly cytoplasmic in control cells and its levels decrease in *put*RNAi without any evidence of nucleolar re-localization (electronic supplementary material, figure S2).

**Figure 6.**
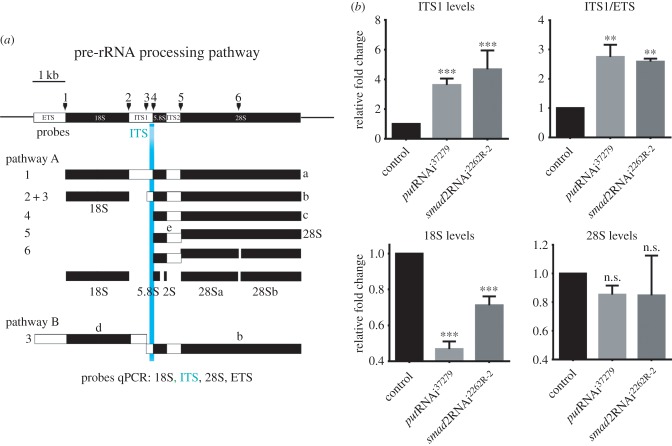
The TGFβ/Activin pathway is required for pre-rRNA processing. (*a*) Diagram showing the several steps of the pre-rRNA processing. (*b*) The relative amounts of the ITS1 (target sequence labelled in light blue), ITS1/ETS ratio, 18S and 28S were measured by qPCR using RNA isolated from control larvae salivary glands or salivary glands from larvae expressing the *put*RNAi or *smad2*RNAi. Data are presented as fold change relative to control and indicate the mean + s.e.m (*n* = 5). Data were normalized to the levels of CaMKII mRNA (n.s. means no statistical difference between samples; ***p < 0.001).

Ultrastructural TEM analysis of salivary gland cells where TGFβ/Activin signalling was inhibited by either *put*RNAi or *smad2*RNAi expression confirmed the presence of nucleolar hypertrophy in these genotypes (figure 5*e–g*). Further, low-contrast intranucleolar regions could be observed, although it is unclear if these regions correspond to the accumulation spots for Vito and RpS6 observed using confocal microscopy (figures 4*f* and 5*d*). Moreover, we detected an accumulation of densely packed particles in the nucleoplasm when compared with control cells (*put*RNAi *n* = 23 out of 23 cells from seven independent salivary glands, figure 5*f–f*″; *smad2*RNAi *n* = 10 out of 18 cells from six independent salivary glands, figure 5*g–g*″). Importantly, only individual particles were found occasionally in controls (figure 5*e*″) and are never found as clusters in our control samples (*n* = 0 out of 16 cells analysed from five independent salivary glands, figure 5*e–e*″). The size of these particles was on the scale expected for pre-ribosomal intermediates undergoing maturation in the path from the nucleolus to the cytoplasm [28]. These particles also resemble the particles found when nucleolar stress was induced in *Drosophila* midgut cells by knockdown of Nopp140 [29]. Thus, our results suggest that ribosome biogenesis had been stalled, in which case we would expect to detect alterations in pre-rRNA processing. The rRNA genes are organized in tandem in several arrays that are transcribed as single units (figure 6*a*) [30]. After being transcribed by RNA polymerase I, the pre-rRNA is subjected to cleavage, 5′ and 3′ exonucleolytic digestion, and base modifications to yield the mature 28S, 18S and 5.8S rRNAs (figure 6*a*) [30,31]. Interestingly, in both *put*RNAi and *smad2*RNAi cells, we detected a strong accumulation of uncleaved pre-rRNA intermediates containing the ITS1 region (figure 6*b*). To further distinguish whether this accumulation is derived from an increased transcription or an accumulation of the uncleaved pre-RNA intermediates, we quantified the relative abundance of pre-rRNA transcripts containing the external transcribed spacer (ETS) region. ETS-containing transcripts are short-lived, as the ETS is the first region to be processed with fast kinetics, and can be used as a proxy for the pre-rRNA transcription rate by the RNA polymerase I. Thus, when the levels of ITS1 were normalized to ETS levels, both TGFβ RNAis present about a threefold increase of ITS1-containing intermediate precursors. Furthermore, the levels of the small ribosome subunit 18S rRNA were also significantly reduced in these cells, whereas no significant differences were detected for the 28S rRNA (figure 6*b*). Together, these results point towards stalled ribosome biogenesis.

### 2.3. Put overexpression exacerbates Myc-induced nucleolar hypertrophy and cell growth

**Figure 7.**
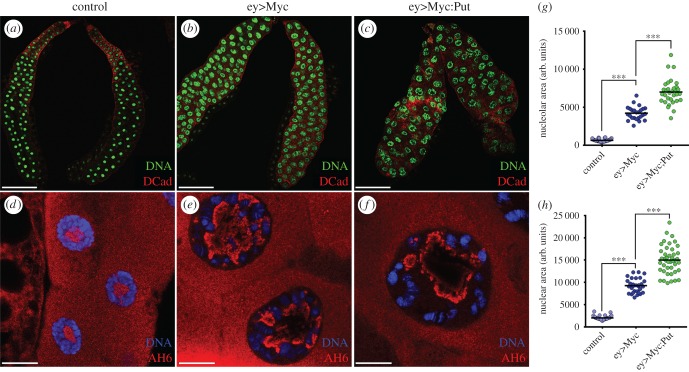
Put overexpression cooperates with dMyc to increase nucleolar hypertrophy and cell growth. (*a–c*) *Drosophila* salivary gland growth is substantially increased by Myc overexpression (ey>Myc) and is enhanced by TGFβ stimulation. (*a*) Lower magnification of the control salivary gland. (*b*) dMyc overexpression results in larger salivary glands with increased nuclear area. (*c*) Ectopic expression of Put potentiates dMyc-induced nuclear overgrowth. Salivary glands of the indicated genotypes were stained for the membrane marker DCad (red) and counterstained with DAPI (green). (*d–f*) Magnification of the nuclei of the indicated genotypes stained with a nucleolar marker AH6 (red) and counterstained with DAPI (blue). (*d*) Control nuclei showing the cytoplasmic and nucleolar localization of the nucleolar marker AH6. (*e*) Nuclear and nucleolar overgrowth induced by dMyc overexpression. (*f*) Nucleolar staining showing the synergistic effect between Put and Myc overexpression. (*g*) Quantification of nucleolar areas after overexpressing dMyc alone or together with Put. (*h*) Quantification of nuclear areas after overexpressing dMyc alone or together with Put. (*n* = 40–65), ***p < 1 × 10^–4^. Scale bars: (*a–c*) 200 µm, (*d–f*) 20 µm.

The ability of Myc to increase ribosome synthesis is an essential mechanism by which Myc promotes both cell growth and proliferation, as well as tumorigenesis [32]. In both mammalian and *Drosophila* cells, this mechanism requires coordination between nucleolar hypertrophy and the stimulation of pre-rRNA transcription and processing [17,18,33–36]. Overexpression of dMyc in salivary gland cells resulted in dramatic increases in nucleolar, nuclear and cellular sizes (figure 7*a,b,d,e,g,h*) [17,37]. dMyc expression also increases ploidy in these cells, an effect that has been proposed to be secondary to the strong stimulation of cell growth [18,38]. It has been shown that the transcription factor E2F1 acts as a ‘growth sensor’ coupling rates of endocycle progression to rates of cell growth [39]. Remarkably, we observed that overexpression of Put significantly enhanced the dMyc-stimulated nucleolar, nuclear and cellular growth (figure 7*c,f,g,h*). The overexpression of Put, on its own, was not sufficient to induce growth in the salivary glands (not shown). In support of these observations, in the eye imaginal disc and resulting adult retinas, the overexpression of Put was also able to synergize with Myc, increasing overall tissue size (electronic supplementary material, figure S3). These results suggest that TGFβ signalling cooperates with dMyc to control nucleolar function and mass accumulation.

## 3. Discussion and conclusions

Taken together, our results show that members of the Activin branch of the TGFβ signalling pathway (the RII receptor Put and the R-Smad Smad2) are autonomously required for cell and tissue growth in the *Drosophila* larval salivary gland. In this simple tissue model, cell growth control can be untangled from cell proliferation and ligand gradient control. Previously, the Activin RI receptor Babo and Smad2 were shown to be specifically required for cellular proliferation and overall growth of the wing imaginal disc [15]. Interestingly, the TGFβ/Activin branch was not found to affect any specific transition of the cell cycle or to cause extensive apoptosis in the wing disc [15]. Recently, TGFβ signalling was also shown to regulate mitochondrial metabolism in *Drosophila* [40], and to promote the Warburg effect (aerobic glycolysis) in breast tumours [41]. These results, together with our previous report of a genetic interaction between members of the TGFβ signalling pathways and *vito*, a nucleolar regulator of growth [16], lead us to focus on the possible regulation of basic mechanisms of cell growth by TGFβ/Activin. We found that interfering with TGFβ/Activin signalling caused changes in nucleolar biogenesis with increased relative areas and altered ultrastructure. Furthermore, this correlated with accumulation of unprocessed intermediate pre-rRNA transcripts, defects in ribosome biogenesis with a significant decrease in 18S rRNA and very significant effects on the nuclear localization of ribosomal proteins. What are the mechanisms by which TGFβ regulates ribosome biogenesis? TGFβ signalling may regulate the transcription of targets with direct enzymatic roles in pre-RNA processing or ribosome biogenesis and nuclear export. The 5′–3′ RNA exonuclease Vito is a strong candidate to fulfil that role. In the budding yeast, the Vito homologue Rrp17p acts as a functional link between late processing of pre-rRNA and nuclear export of pre-60S ribosomal subunits [42]. When we interfered with Put function, Vito levels increased, but Vito accumulated in intranucleolar spots and thus might not be available at the peripheral nucleoplasm to efficiently chaperone pre-ribosomal particles for nuclear export. That would explain the observed accumulation of RpL10A and RpL22 (and putative ribosomal particles detected by TEM) at the peripheral nucleoplasm. At the same time, Rrp17p is required for ITS1 processing [42,43], thus the misregulation of Vito function in salivary gland cells could cause the observed increase in pre-rRNA intermediate transcripts, and the observed accumulation of RpS6 in intranucleolar spots.

Could TGFβ regulate ribosome biogenesis through other novel mechanisms? In fact, in breast cancer cells, a cancer-specific nuclear translocation of TβRI was shown to regulate nuclear mRNA processing [44]. Furthermore, in mammalian cells, TGFβ signalling has also been shown to regulate directly the biogenesis of a set of miRNA at the post-transcriptional level [45,46]. Surprisingly, R-Smads associated with the large Drosha/DGCR8/p68 microprocessor complex have been shown to bind pri-miRNAs and facilitate the cleavage of pri-miRNA to pre-miRNA by Drosha [45,46]. Alternatively, TGFβ may regulate the expression or nucleolar recruitment of ribosomal proteins, causing changes in nucleolar dynamics and indirectly affecting pre-rRNA processing. In fact, in Diamond–Blackfan anaemia, haploinsufficiency for several ribosomal genes has been shown to affect pre-ribosomal RNA (pre-rRNA) processing and thus to interfere with ribosome biogenesis [47]. Despite the precise mechanism, we show for the first time that TGFβ/Activin signalling is required for normal assembly of the nucleolus and pre-rRNA processing.

## 4. Material and methods

### 4.1. Fly strains and husbandry

All crosses were raised at 25°C under standard conditions and for synchronization; all the conditions were analysed after a single day of egg collection. The following stocks (described in FlyBase, unless stated otherwise) were used: ey-Gal4, UAS-*lacZ* and the wild-type strain w1118. UAS*-CD4tdTomato* was used to report salivary glands expression of ey-Gal4 (electronic supplementary material, figure S4*a–c*′). *AB1*-Gal4 and *ptc*-Gal4 were used as salivary gland alternative drivers with similar results to ey-Gal4 (electronic supplementary material, figure S4*d–f*′). The TGFβ RNAis were obtained from different collections: *put*RNAi#37279 (VDRC), *put*RNAi 7904R-3 (Nigfly), *smad2*RNAi (#2262R-2, Nigfly) and *mad*RNAi (#31315, TRiP). The following TGFβ pathway mutants were obtained from the Bloomington Stock Center: *put*^135^, *put*^10460^, *babo*^32^, *babo*^K16912^, *tkv*^1^ and *tkv*^8^. Overexpression studies were done using UAS-Put [48] and UAS-dMyc [49]. The protein trap strains used in these studies were RpL41YFP (#115-344 Cambridge Protein Trap YFP insertions) and RpS9YFP (#115-034 Cambridge Protein Trap YFP insertions).

### 4.2. Immunostaining

Eye-antennal imaginal discs and salivary glands were prepared for immunohistochemistry using standard protocols. As the growth conditions strongly affect salivary gland size, all the experiments were controlled by synchronization of L3 wandering larvae after a single-day egg collection. To further control this issue, a controlled the egg laying for 5 h was set up, and the salivary glands were analysed 96 h after egg laying (96–101 h AEL; electronic supplementary material, figure S4*g–j*′).

Primary antibodies used were: mouse anti-Armadillo N27A1 at 1 : 100 (Developmental Studies Hybridoma Bank, DSHB), mouse anti-Dlg at 1 : 1000 (4F3, DSHB), rabbit anti-Viriato (Vito) at 1 : 250 (ABGent), rat anti-DCad at 1 : 100, mouse anti-AH6 at 1 : 10 (DSHB), rabbit anti-Fibrillarin at 1 : 250 (Abcam, #ab5821), mouse anti-Fibrillarin at 1 : 500 (Abcam, #ab4566), mouse anti-RpS6 at 1 : 100 (Cell Signaling, #2317), mouse anti-RpL11 at 1 : 100 (Abcam, #ab79352), mouse anti-RpL10A at 1 : 400 (Abcam, #ab55544), rabbit anti-RpL22 at 1 : 100 (kind gift from Dr Vassie Ware). To stain for cellular limits phalloidin conjugated with rhodamine was used at a dilution of 1 : 1000. Appropriate Alexa-Fluor conjugated secondary antibodies were from Molecular Probes. Images were obtained with the Leica SP2 confocal system or Leica SP5 confocal system and processed with Adobe Photoshop.

### 4.3. Size measurements and statistics

Salivary gland areas were measured using the Polygon selection tool of ImageJ 1.48r software (NIH, Bethesda, MA), considering the limits stained by Arm, Dcad or RhPh and represented as arbitrary units. The cellular parameters shown in this study were measured using the Polygon selection tool of ImageJ 1.48r. The nucleolar area was determined using the nucleolar markers RpL41YFP, anti-AH6 or anti-Fibrillarin and DAPI staining was used to stain for the nuclear area. The results are presented as the ratio of the nucleolar area to the nucleus that it corresponds to. The intensity of the nucleolar components was determined using a fixed ROI circle, and the mean intensity of each nucleolus was measured using ImageJ. To each measurement, another nucleolar component was used as reference (for example, AH6 and Fibrillarin). For each genotype, five to six nuclei from the proximal region of at least five to six independent salivary glands were used. Statistical analysis and generation of the graphical output was done using GraphPad Prism v. 5.0. Statistical significance was determined using an unpaired, two-tailed Student's *t*-test, with a 95% confidence interval, after assessing the normality distribution of the data with D'Agostino–Pearson normality test.

### 4.4. Transmission electron microscopy

Dissected third-instar salivary glands were fixed with 2.5% glutaraldehyde in 0.1 M sodium cacodylate buffer for 30 min and post-fixed with 4% osmium tetroxide. After washing, salivary glands were incubated with 0.5% uranyl acetate (30 min) and further dehydrated through a graded ethanol series (70% for 10 min, 90% for 10 min and four changes of 100%). Salivary glands were then soaked in propylene oxide for 10 min and then in a mixture (1 : 1) of propylene oxide and Epon resin (TAAB Laboratories) for 30 min. This mixture was then replaced by 100% Epon resin for 24 h. Finally, fresh Epon replaced the Epon and polymerization took place at 60°C for 48 h. Ultrathin sections were obtained using an ultramicrotome, collected in copper grids and then double contrasted with uranyl acetate and lead citrate. In total, at least 16 independent cells of five independent salivary glands were analysed for each genotype. Micrographs were taken using a TEM Jeol JEM-1400, with Orius SC 1000 digital camera (80 kV).

### 4.5. Quantitative real-time PCR

For qPCR experiments, all the RNAis were induced with the ey-Gal4 driver, and the salivary glands of wandering L3 instar larvae were dissected. The number of salivary glands was determined according to its size to yield similar RNA concentrations (i.e. for w1118 control strain, a minimum of 30 salivary glands were dissected, for ey-Gal4; *put*RNAi^37279^ 50–60 salivary glands were dissected and 40–50 salivary glands were dissected for ey-Gal4; *smad2*RNAi^2262R−2^).

The RNA was extracted using TRIzol (Invitrogen) according to the manufacturer's instructions and treated with Turbo DNase I (Ambion). cDNA was generated by reverse transcription with the SuperScript III First-Strand Synthesis SuperMix for qRT-PCR (Invitrogen). Quantitative real-time PCR analysis was performed in triplicate in 20 µl reactions containing iQ SYBR Green Supermix (BioRad), each gene-specific primer at 250 nM and 1 µl of cDNA template. Cycling conditions in a BioRad iQ5 instrument were 95°C for 3 min, followed by 40 cycles of denaturation at 95°C for 10 s and annealing for 30 s at 53°C, 60°C or 64°C depending on the primer set. Fold change relative to the expression of *CaMKII*, which has been used previously as a control for gene expression [17], was calculated using the 2^–ΔCT^ method [50]. Three to five biological replicates were analysed for each primer set. The following primer pairs (from 5′ to 3′) were used:

*CaMKII* (control): *Fw*—TTACACCATCCCAACATAGTGC

*Rev*—CAAGGTCAAAAACAAGGTAGTGATAG;

28S: *Fw*—GGAGGATCTTCGATCACCTGATG

*Rev—*GCTGCTCAACCACTTACAACAC;

18S: *Fw—* TGGTCTTGTACCGACGACAG

*Rev*—GCTGCCTTCCTTAGATGTGG;

ITS1: *Fw*—TTATTGAAGGAATTGATATATGCC

*Rev*—ATGAGCCGAGTGATCCAC;

ETS: *Fw*—GCTCCGCGGATAATAGGAAT

*Rev*—ATATTTGCCTGCCACCAAAA.

## Supplementary Material

Figure S1; Figure S2; Figure S3; Figure S4; Supplementary Figure legends

## Data accessibility

The datasets supporting this article have been uploaded as part of the electronic supplementary material.
