# Supplementary material for: TGFβ/Activin signalling is required for ribosome biogenesis and cell growth in Drosophila salivary glands
Source: Open Biol. 2017 Jan 25;7(1):160258. doi: 10.1098/rsob.160258 (PMC5303274; doi:10.1098/rsob.160258)
Supplement: Figure S1; Figure S2; Figure S3; Figure S4; Supplementary Figure legends [file rsob160258supp1.pdf]

Online Supplementary Material

Open Biology – DOI - 10.1098/rsob.20160258

Title: TGFβ/Activin signalling is required for ribosome biogenesis and cell growth in Drosophila salivary glands

Torcato Martins[1,2,3#], Nadia Eusebio[1,2], Andreia Correia[1,2], Joana Marinho[1,2], Fernando Casares[4] & Paulo S. Pereira[1,2#]

[1]I3S - Instituto de Investigação e Inovação em Saúde, Universidade do Porto, Portugal. [2]Instituto de Biologia Molecular e Celular (IBMC), Universidade do Porto. Porto 4150-180, Portugal. [3]Cell Cycle Development Group, Department of Genetics, University of Cambridge, Downing Street, Cambridge CB2 3EH, UK. [4]Centro Andaluz de Biología del Desarrollo (CABD), CSIC-JA-Universidad Pablo de Olavide. Ctra. de Utrera km1, Seville 41013, Spain.

Figure S1 – Eye and salivary gland phenotypes induced by RNAi for TGF-β signalling pathways.

(A-C) *put* depletion impairs photoreceptors differentiation: **(A)** Control eye imaginal disc (*ey*>lacZ). **(B)** *put*RNAi$^{7904R\text{-}3}$ induction results in smaller imaginal discs with a severe delay in morphogenetic furrow progression. **(C)** Strong depletion of *put* affects eye imaginal disc growth and completely abolishes photoreceptor differentiation in L3 imaginal discs. The eye imaginal discs were stained with DAPI (green) and D-Cadherin (red) to label nuclei and cell membrane respectively. **(D-F)** 3rd instar larvae eye-antennal imaginal discs of R-Smad RNAi depletion genotypes: **(D)** Control eye imaginal disc (*ey*>lacZ). **(E)** *smad2*RNAi slightly affects eye imaginal disc size (*ey>smad2*RNAi). **(F)** *mad*RNAi strongly impairs eye primordia growth (*ey>mad*RNAi). The imaginal discs were labelled for DNA using DAPI(green) and Armadillo for cell limits (red). **(G-J)** Salivary glands of the R-Smads depletions: **(G)** Control salivary gland. (**H**) *smad2*RNAi salivary glands are smaller than controls. (**I**) Absence of *mad* does not affect salivary glands size. (**J**) Co-depletion of *smad2* and *mad* resembles the *smad2* depletion phenotype. All the salivary glands were stained with DAPI (green) and the limits are depicted by a red dashed line. Scale bars: A-F = 50 μm G-J = 200 μm

Figure S2 - Inhibition of TGF β /Activin signalling activity by *put*RNAi causes a decrease in RpS9 levels.

Control salivary glands show cytoplasmic localisation of RpS9YFP and a weak accumulation of RpS9 at the nucleolus (left panels). In *put* depleted salivary glands, RpS9YFP signal is severely reduced at the cytoplasm (right panels). Both conditions

were stained with DAPI to label the nuclear area. Scale bars: Top panels = 50 μm, Bottom panels = 20 μm

Figure S3 – Overexpression of Punt synergises with Myc in retinal growth.
Adult retinas from flies of the indicated genotypes (upper panel). Eye imaginal discs were stained with DAPI (red) and Elav (green) to label nuclei and photoreceptors respectively (lower panels). Scale bar corresponds to 50 μm

Figure S4 – Salivary gland expression controls. (A-C) *ey*-Gal4 driven expression of a membrane marker CD4-tdTomato. *ey*-Gal4 expression in salivary glands starts at the beginning of larval development L1 **(A-A')** and is sustained during the subsequent larval stages, L2 and L3 **(B-C'). A-C'** were counterstained with DAPI to label the DNA and Dlg to reveal the cellular membrane. (D-F') Depletion of *put* with other salivary gland drivers result in a similar decrease in cellular size and an expansion of the nucleolar size. **(D-D')** *ptc*>lacZ presents a nucleolar size similar to wild type strains. *ptc*Gal4-driven *put* depletion results in smaller salivary glands **(E)** with an expansion on the nucleolar area (**E'**, Fib in green labels the nucleolar area). A similar phenotype is obtained when *put* is depleted using the AB1-Gal4 driver (**F,F'**, AH6 in green labels the nucleolar area). **(G-J')** TGFβ/Activin decreased activity causes growth deficits in synchronised larvae after restricted egg laying interval (96h-101h AEL). **(G-G')** Control salivary glands were dissected 96h to 101h after egg laying and stained for the nucleolus (Fib, green) and cellular membrane (RhPh, red). Depletion of *put* **(H-I')** or *smad2* **(J-J')** in restricted collections results in a growth deficit with expansion of the nucleolar size as presented in figure **1** and **2**. Scale bars: A-F, G'-J' = 50 μm; A'-F' = 20 μm; G-J = 200 μm

FIGURE S1

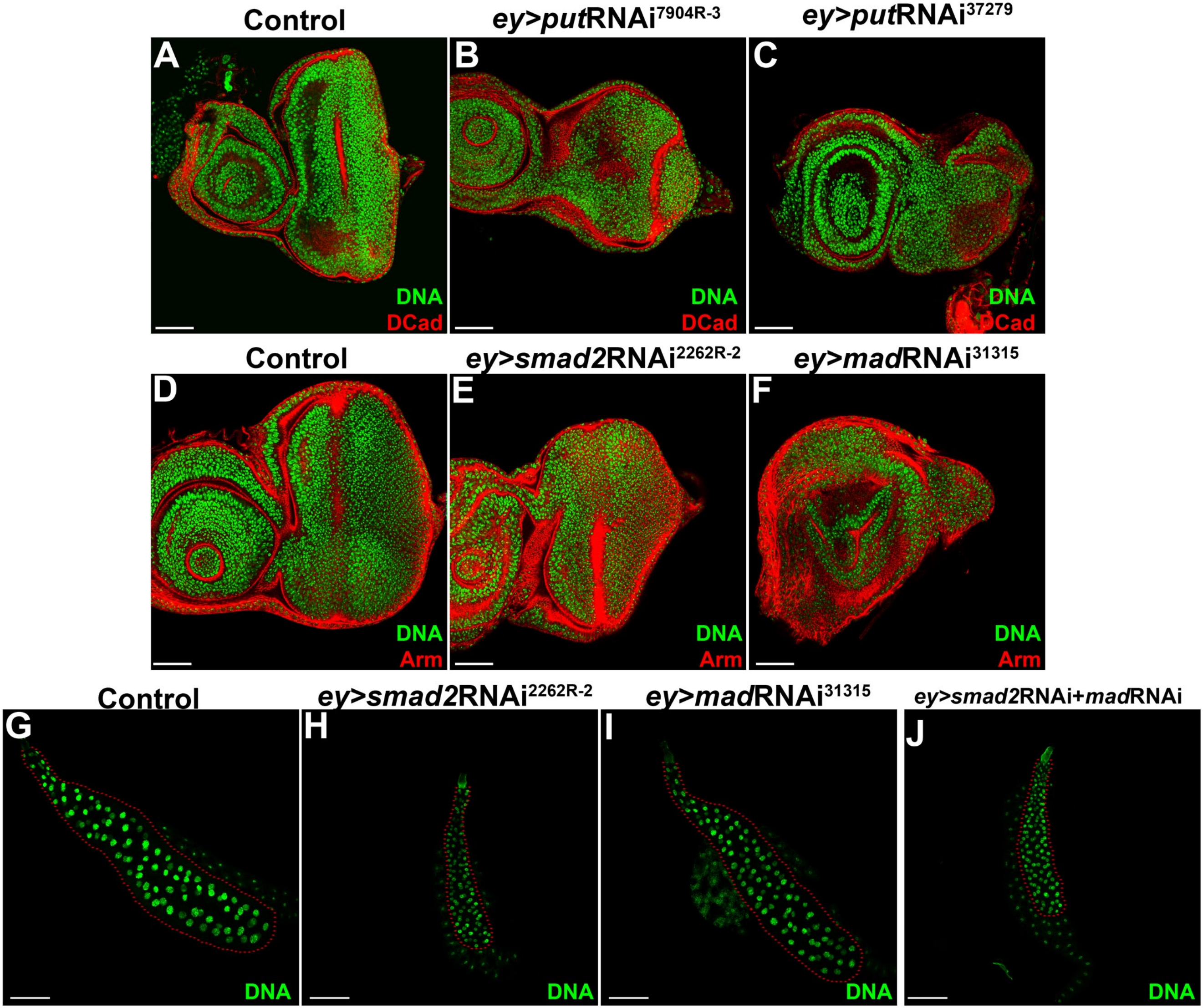
Control
ey>putRNAi7904R-3
ey>putRNAi37279
A
B
C
DNA
DCad
Control
ey>smad2RNAi2262R-2
ey>madRNAi31315
D
E
F
DNA
Arm
Control
ey>smad2RNAi2262R-2
ey>madRNAi31315
ey>smad2RNAi+madRNAi
G
H
I
J
DNA

FIGURE S2

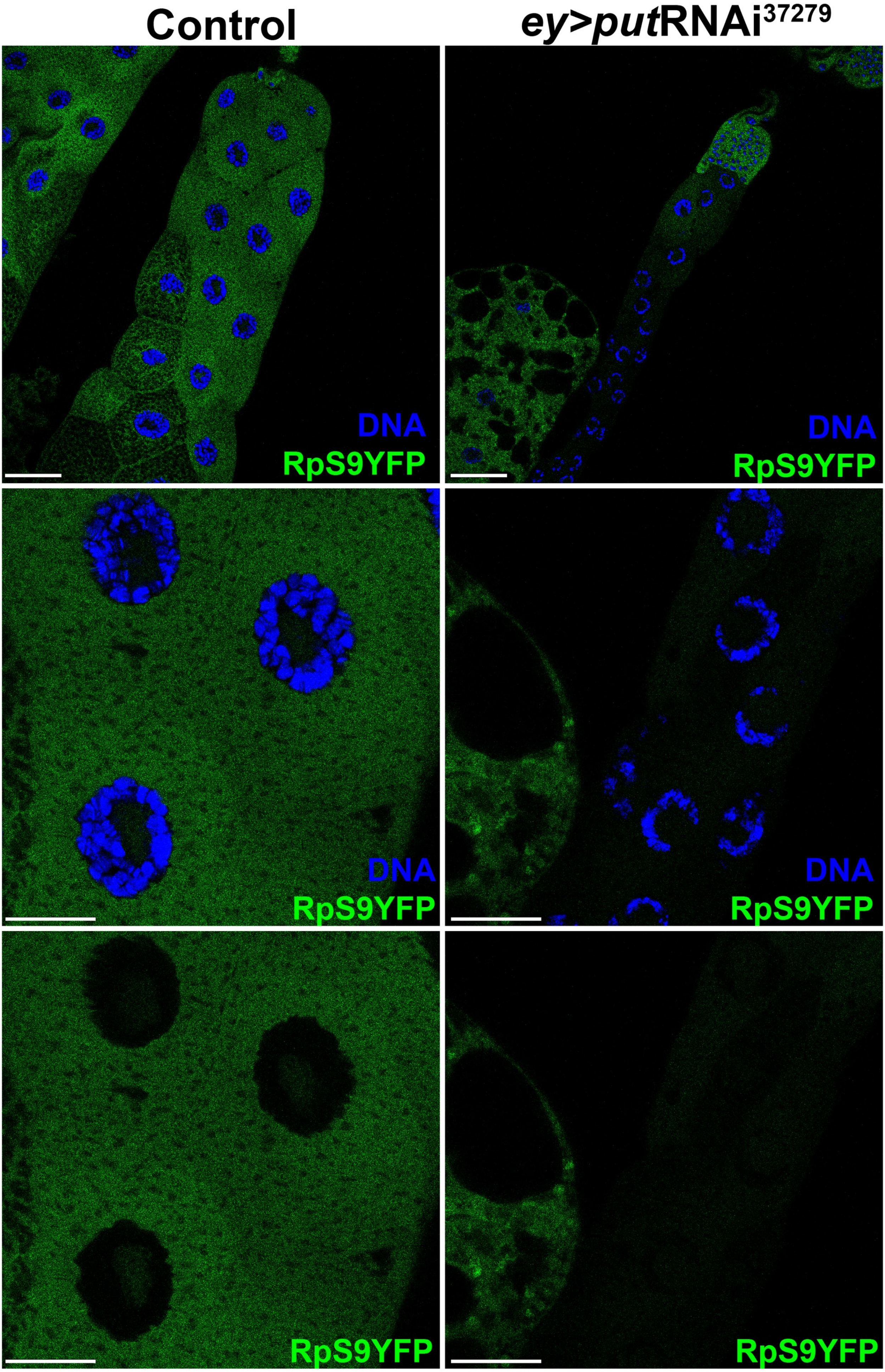
Control
ey>putRNAi37279
DNA
RpS9YFP
DNA
RpS9YFP
DNA
RpS9YFP
DNA
RpS9YFP
RpS9YFP
RpS9YFP

FIGURE S3

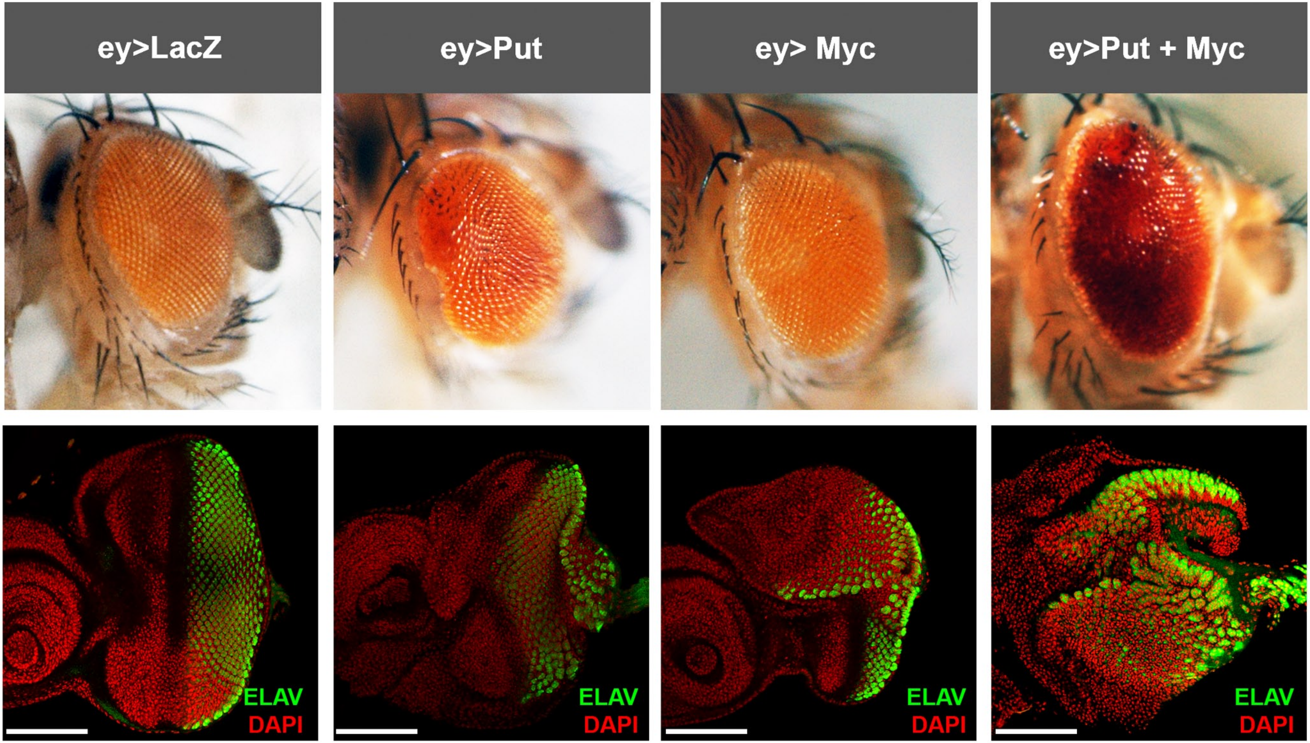
ey>LacZ
ey>Put
ey> Myc
ey>Put + Myc
ELAV
DAPI
ELAV
DAPI
ELAV
DAPI
ELAV
DAPI

FIGURE S4

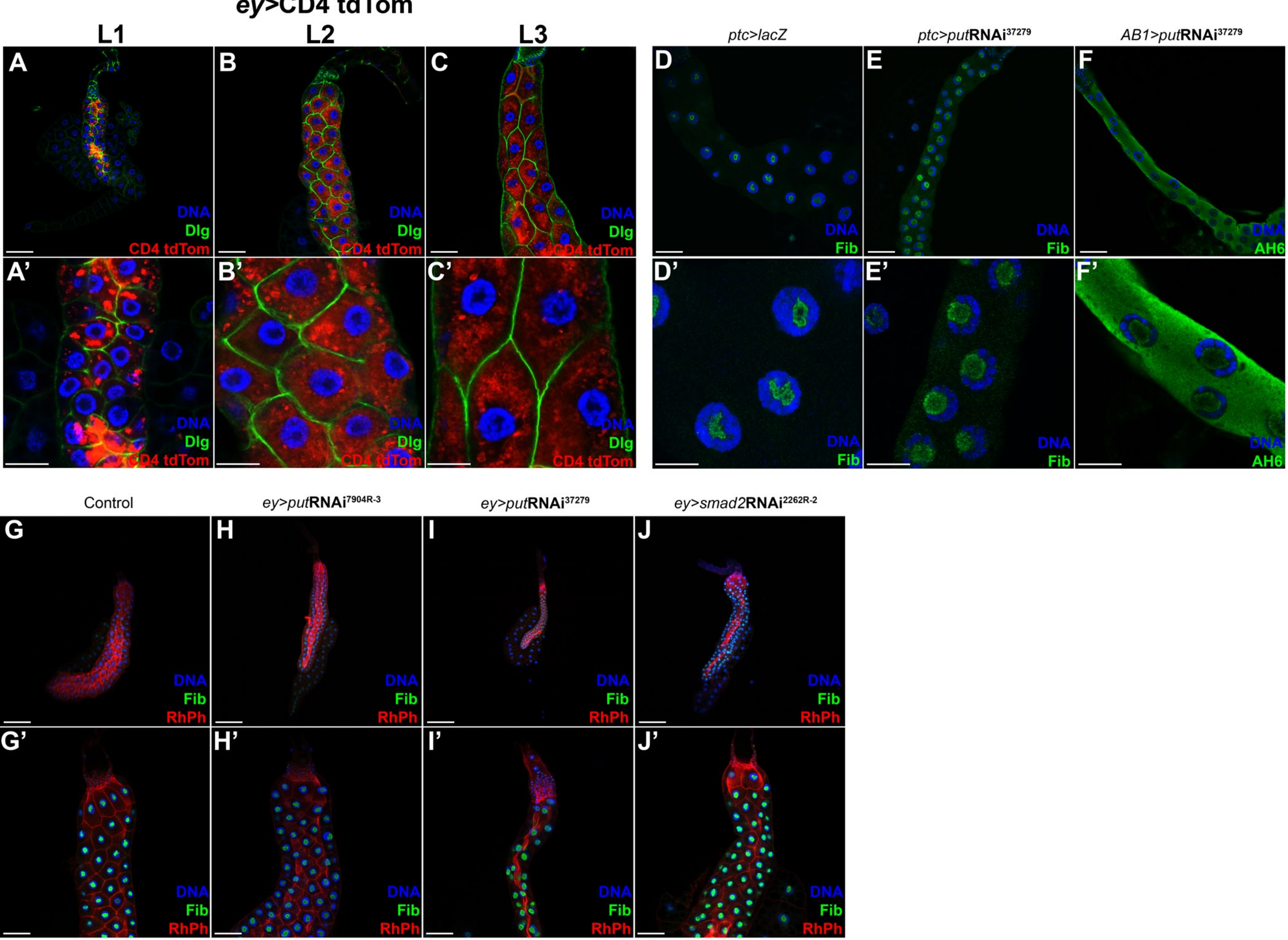

ey>CD4 tdTom
L1
L2
L3
ptc>lacZ
ptc>putRNAi37279
AB1>putRNAi37279
A
B
C
D
E
F
DNA
Dlg
CD4 tdTom
Fib
AH6
A'
B'
C'
D'
E'
F'
Control
ey>putRNAi7904R-3
ey>putRNAi37279
ey>smad2RNAi2262R-2
G
H
I
J
RhPh
G'
H'
I'
J'
